# Deciphering white matter microstructural alterations in catatonia according to ICD-11: replication and machine learning analysis

**DOI:** 10.1038/s41380-024-02821-0

**Published:** 2024-12-02

**Authors:** Robin Peretzke, Peter F. Neher, Geva A. Brandt, Stefan Fritze, Sebastian Volkmer, Jonas Daub, Georg Northoff, Jonas Bohn, Yannick Kirchhoff, Saikat Roy, Klaus H. Maier-Hein, Andreas Meyer-Lindenberg, Dusan Hirjak

**Affiliations:** 1 Division of Medical Image Computing, German Cancer Research Center, Heidelberg, Germany; 2 Medical Faculty, Heidelberg University, Heidelberg, Germany; 3 Pattern Analysis and Learning Group, Department of Radiation Oncology, Heidelberg University Hospital, Heidelberg, Germany; 4 German Cancer Consortium (DKTK), Heidelberg, Germany; 5 Department of Psychiatry and Psychotherapy, Central Institute of Mental Health, Medical Faculty Mannheim, Heidelberg University, Mannheim, Germany; 6 German Center for Mental Health (DZPG), partner site Mannheim-Heidelberg-Ulm, Mannheim, Germany; 7 Hector Institute for Artificial Intelligence in Psychiatry, Central Institute of Mental Health, Medical Faculty Mannheim, Heidelberg University, Mannheim, Germany; 8 Mind, Brain Imaging and Neuroethics Research Unit, Institute of Mental Health Research, Faculty of Medicine, University of Ottawa, Ottawa, ON Canada; 9 Faculty of Bioscience, Heidelberg University, Heidelberg, Germany; 10 National Center for Tumor Diseases (NCT), NCT Heidelberg, DKFZ and University Medical Center Heidelberg, Heidelberg, Germany; 11 Faculty of Mathematics and Computer Science, Heidelberg University, Heidelberg, Germany; 12 HIDSS4Health - Helmholtz Information and Data Science School for Health, Karlsruhe/Heidelberg, Germany

**Keywords:** Neuroscience, Diagnostic markers

## Abstract

Catatonia is a severe psychomotor disorder characterized by motor, affective and cognitive-behavioral abnormalities. Although previous magnetic resonance imaging (MRI) studies suggested white matter (WM) dysconnectivity in the pathogenesis of catatonia, it is unclear whether microstructural alterations of WM tracts connecting psychomotor regions might contribute to a better classification of catatonia patients. Here, diffusion-weighted MRI data were collected from two independent cohorts (whiteCAT/replication cohort) of patients with (n = 45/n = 13) and without (n = 56/n = 26) catatonia according to ICD-11 criteria. Catatonia severity was examined using the Northoff (NCRS) and Bush-Francis (BFCRS) Catatonia Rating Scales. We used tract-based spatial statistics (TBSS), tractometry (TractSeg) and machine-learning (ML) to classify catatonia patients from tractometry values as well as tractomics features generated by the newly developed tool RadTract. Catatonia patients showed fractional anisotropy (FA) alterations measured via TractSeg in different corpus callosum segments (CC_1, CC_3, CC_4, CC_5 and CC_6) compared to non-catatonia patients across both cohorts. Our classification results indicated a higher level of performance when trained on tractomics as opposed to traditional tractometry values. Moreover, in the CC_6, we successfully trained two classifiers using the tractomics features identified in the whiteCAT data. These classifiers were applied separately to the whiteCAT and replication cohorts, demonstrating comparable performance with Area Under the Receiver Operating Characteristics (AUROC) values of 0.79 for the whiteCAT cohort and 0.76 for the replication cohort. In contrast, training on FA tractometry resulted in lower AUROC values of 0.66 for the whiteCAT cohort and 0.51 for the replication cohort. In conclusion, these findings underscore the significance of CC WM microstructural alterations in the pathophysiology of catatonia. The successful use of an ML based classification model to identify catatonia patients has the potential to improve diagnostic precision.

## Introduction

Catatonia is characterized by specific psychomotor symptom patterns, that is, specific constellations of decreased (staring, ambitendency, negative, stupor, mutism, etc.), increased (hyperactivity, aggression, impulsivity, combativeness, etc.) or abnormal (mannerism, posturing, stereotypy, verbigeration, waxy flexibility, etc.) psychomotor activity [1–6]. From a nosological perspective, catatonia can be diagnosed according to DSM-5 and ICD-11 classification systems and is a paradigmatic example for a group of psychomotor abnormalities which occurs in a trans-diagnostic way across different psychiatric disorders [6–8]. Catatonia is reported in 5–18% of patients in inpatient psychiatric units and 3.3% on neurology/neuropsychiatric tertiary care inpatient units [9]. In the context of routine clinical practice, catatonia needs to be identified and treated as soon as possible because patients in this state are more susceptible to secondary complications like pneumonia, malnourishment, decubitus, electrolyte imbalances, and dehydration [10]. The predominant focus of the available treatment options is on well-established mechanisms of action, specifically GABAergic, glutamatergic, and dopaminergic transmission [11].

On a neuronal level, studies using magnetic resonance imaging (MRI) have suggested a pathophysiological model of catatonia that involves abnormalities in different cortical and subcortical brain regions, namely in the orbitofrontal cortex (OFC), prefrontal cortex (PFC), supplementary motor area (SMA), primary motor cortex (M1), basal ganglia (BG), thalamus (THAL), amygdala, corpus callosum (CC), cerebellum and brainstem, respectively [12]. This said, catatonia relies on a highly complex interaction between non-motor (e.g., orbitofrontal-affective and default-mode networks) and motor (e.g., the dopaminergic-based subcortical-cortical motor circuit) brain networks and neurotransmitters. Furthermore, dysconnectivity of large-scale neural networks underlying catatonia may be linked to aberrant white matter (WM) microstructure. This assertion is supported by at least three lines of evidence: First, a number of case studies have demonstrated that WM abnormalities can cause catatonia when combined with immune dysregulation [13]. Second, Hagemeyer et al. [14] found a relationship between Cnp partial loss-of-function and catatonia-depression syndrome in both mice and humans. The Ccp gene (chr17q21.2, 11Kb) is responsible for producing the enzyme 2′,3′-cyclic nucleotide 3′-phosphodiesterase (Cnp) [14]. Cnp makes up approximately 4% of all myelin proteins in the central nervous system and has been found in specific regions of non-compacted myelin, including the inner mesaxon, paranodal loops, and Schmidt-Lantermann incisures [15, 16]. The results of Hagemeyer et al. [14] and Janova et al. [17] also supported the idea that late-onset low-grade inflammation in mice is a possible mechanism of catatonia-depression syndrome [14, 17]. Furthermore, both studies [14, 17] observed a strong correlation between the presence of severe catatonic signs and the CNP partial loss-of-function genotype rs2070106-AA in two separate schizophrenia groups. According to the study by Hagemeyer et al. [14], neuroimaging could identify axonal degeneration in the frontal corpus callosum of individuals with the low-expression genotype (CNP rs2070106-AA), indicating a similar mechanism. Third, four diffusion MRI (dMRI) studies showed a significant association between WM alterations and catatonia [18–21]. In particular, among these dMRI studies, the role of CC in the pathogenesis of catatonia was often discussed because it plays a crucial role in the interhemispheric transfer of information [18, 22]. A very recent multimodal anatomical study [23] showed significant differences in CC between SSD and BD patients compared to healthy controls, particularly in the anterior body and isthmus for SSD, and solely in the isthmus for BD. Given that these sub-regions connect the prefrontal, temporal, and inferior parietal cortical areas involved in specific affective, sensorimotor and cognitive functions, alterations in the shape and size of these areas could partly contribute to catatonia signs. Consequently, these sub-regions of the CC may hold diagnostic relevance for catatonia. However, none of the previous dMRI studies diagnosed catatonia according to ICD-11, tried to replicate their results in an independent patient sample and applied multiple analysis methods including machine-learning (ML) based classification approaches on both, the well-established tractometry features as well as features extracted by the newly developed tractomics technique.

To overcome the aforementioned bias, the present study had two main objectives: First, we examined two independent cohorts of patients diagnosed with catatonia according to ICD-11, along with two age- and gender-matched control groups consisting of patients with mental disorders other than catatonia, using dMRI. Second, we used a combination of three distinct diffusion-tensor imaging (DTI) analysis techniques to examine microstructural alterations in WM tracts underlying catatonia. In particular, we used a whole-brain voxel-wise analysis of fractional anisotropy (FA), medial diffusivity (MD) and radial diffusivity (RD) in catatonia using Tract-Based Spatial Statistics (TBSS) [24]. Then, we used TractSeg [25], which allowed an accurate reconstruction of fiber tracts in subject space, thus avoiding the problem of inaccurate coregistration of tracts with varying size and shape. We performed statistical analyses by evaluating FA, MD, RD along neurobiologically plausible tracts (tractometry) which represent important connections between catatonia-associated brain regions identified in previous studies such as OFC, PFC, SMA, M1, BG, THAL, CC, cerebellum and brainstem. Therefore, we have opted for the following 15 tracts as mentioned in Wasserthal et al. [25, 26]: CC [Rostrum (CC 1), Genu (CC 2), Rostral body (CC 3), Anterior midbody (CC 4), Posterior midbody (CC 5), Isthmus (CC 6), and Splenium (CC 7)], corticospinal tract (CST), thalamo-premotor (T_PREM), striato-fronto-orbital (ST_FO), and striato-premotor (ST_PREM). Finally, we employed a machine learning (ML) approach to categorize patients with catatonia according to ICD-11 using different types of tractspecific features. This involved utilizing features generated by TractSeg (FA, MD, RD), alongside a more extensive range of radiomics features (tractomics) produced by a newly developed tool called RadTract (https://github.com/MIC-DKFZ/RadTract) [27]. RadTract is a novel method developed by Neher et al. [27] that enhances tract-specific analysis of WM using diffusion MRI by integrating radiomics-based imaging features, enabling improved predictive modeling while preserving the localization capability of tractometry. Traditional tractometry methods rely on summary statistics, which may overlook valuable information, limiting their applicability for predictive modeling. In contrast, RadTract extracts a rich representation of image information along tracts by subdividing each WM tract into parcels and calculating a comprehensive set of quantitative features from diffusion parameter maps. This approach, combining tractometry with radiomics, allows for detailed analysis of variations within the WM tract and localization of informative regions, demonstrating superior performance in diagnosing disease subgroups and estimating demographic and clinical parameters.

In particular, this study tested three main hypotheses: First, we hypothesized that we will find differences in WM tracts connecting psychomotor regions between patients with and without catatonia according to ICD-11. Second, we hypothesized that WM microstructural alterations of tracts connecting psychomotor regions can serve as training data for machine learning classifiers to differentiate between patients with and without catatonia according to ICD-11. Third, we hypothesized that WM microstructural alterations will be associated with catatonia severity, and we will be able to replicate these findings in a different cohort of patients with and without catatonia according to ICD-11.

## Experimental procedures

### Study participants

In this study, we included two independent cohorts of patients with and without catatonia according to ICD-11 stemming from two different projects conducted at Central Institute of Mental Health (CIMH) in Mannheim, Germany:

The whiteCAT cohort consisted of 46 right-handed [28] subjects fulfilling the ICD-11 criteria for catatonia associated with another mental disorder (6A40) and 56 psychiatric controls (schizophrenia or other primary psychotic disorder and mood disorders) without catatonia according to ICD-11. The study participants in the whiteCAT cohort had been recruited between April 2022 and February 2024 and used in previous studies of our group [29, 30]. Psychiatric diagnoses have been made by the German Mini Diagnostic Interview for Mental Disorders (Mini-DIPS) (GAB, JD, SF and DH) [31].

The replication cohort consisted of 13 right-handed [28] subjects fulfilling the ICD-11 criteria for catatonia associated with another mental disorder (6A40) and 26 psychiatric controls (schizophrenia or other primary psychotic disorder) without catatonia according to ICD-11. The study participants in the replication cohort had been recruited between February 2017 and October 2018 and used in previous studies of our group [32–34]. Diagnoses were made by staff psychiatrists and confirmed using the German versions of the Structured Clinical Interview for DSM-IV-TR axis I and II disorders (SCID) and examination of the case notes (DH and SF).

The local Ethics Committees I and II (Medical Faculty Mannheim and Medical Faculty Heidelberg at Heidelberg University, Germany) approved the studies. We obtained written informed consent from all study participants after all aims and procedures of the study had been fully explained.

### Clinical assessment

Both cohorts were examined during in- or outpatient treatment at CIMH within one week after partial remission of acute psychopathological symptoms or during outpatient treatment, but by different examiners (whiteCAT: GAB, SV and JD; replication cohort: SF and DH). All relevant study procedures (e.g. psychopathological rating scales, neuropsychological assessments, sensorimotor/catatonia assessment and MRI examination) were completed within 7 days. All patients were on a stable daily dose of antipsychotic or antidepressant medication for at least 7 days. To investigate the effect of antipsychotics and antidepressant medication on results, in the whiteCAT cohort, antipsychotic and antidepressant medication were standardized as Olanzapine [OLZe] [35] and fluoxetine [FLXe] [36] equivalents. OLZe and FLX equivalents were z-transformed, summed up and included as covariates (MED) in subsequent analyses. In the replication cohort, all patients were exclusively on antipsychotic medication, standardized to Olanzapine (OLZe) [35], which was included as a covariate in subsequent analyses. For details on the individual psychotropic agents see Supplementary Material. For the assessment of catatonia signs we used the German version of the Northoff Catatonia Rating Scale (NCRS) [37]. The scale measures the presence and severity of motor (13 items), affective (12 items) and behavioral (15 items) signs in catatonia and therefore comprises three domains, i.e. motor, affective and behavioral domain. According to ICD-11, for the diagnosis of catatonia the presence of three or more of the following signs of decreased (e.g. staring, ambitendency, negativism, stupor, and mutism), increased (e.g. extreme hyperactivity or agitation for no reason with non-purposeful movements and/or uncontrollable, extreme emotional reactions, impulsivity or combativeness), or abnormal (e.g. grimacing, mannerisms, posturing, stereotypy, rigidity, echophenomena, verbigeration, waxy flexibility and catalepsy) psychomotor activity is required. When assessing increased psychomotor activity, multiple phenomena of increased psychomotor activity are counted as one of the required three catatonia signs. It should be noted that the ICD-11 has been available to everyone since its introduction on January 1^st^, 2022. However, the draft of the ICD-11 in German is unusable for official purposes for licensing reasons. For the whiteCAT study, we therefore carefully translated the English ICD-11 into German and compared it with the items of the NCRS. While the NCRS contains 40 catatonia signs, only 23 catatonia signs are listed in the ICD-11. Therefore, only these 23 catatonia signs were taken into account when making an ICD-11 diagnosis. As the English version of the ICD-11 does not contain specific guidelines for the assessment and duration of catatonia signs, we used the NCRS criteria as a guide.

For the examination of other psychomotor abnormalities, we employed the following rating scales: parkinsonism: Simpson and Angus Scale (SAS) [38]; akathisia: Barnes Akathisia Rating Scale (BARS) [39, 40]; and tardive dyskinesia: Abnormal Involuntary Movement Scale (AIMS) [41]. The evaluation of psychopathology and global functioning was performed with Positive and Negative Syndrome Scale (PANSS) [42], Clinical Global Impression Scale (CGI) [43] and Global assessment of functioning (GAF) [44].

Eligible patients in the psychiatric control groups in both cohorts were all adults aged between 18 and 64 with the ICD-11 diagnosis of schizophrenia or other primary psychotic disorders (6A20, 6A21, 6A22, 6A23, 6A24, 6A25) or mood disorder (6A60-6A62, 6A70-6A73) with or without psychotic symptoms. Patients in the control group were not allowed to meet the diagnostic criteria of catatonia according to ICD-11. Additionally, an important aim was to ensure that patients in the control groups of both cohorts had no previous (recurrent) catatonic episodes. To this end, we obtained a written permission of the patients to use CIMH health records and, where possible, the patients’ external health records. That is, patients with previous (recurrent) catatonic episodes who did not currently fulfil the ICD-11 criteria for catatonia were excluded from both cohorts.

Exclusion criteria were: inability to speak German, known intellectual disability (IQ < 70) or dementia, history of alcohol or drug dependence in remission for less than 12 months, and history of neurological disease or medical conditions that could impact the measurement of the constructs being assessed. In the whiteCAT cohort, history of alcohol or drug dependence was ruled out thorough clinical interview and the MINI-DIPS diagnostic interview. In the replication cohort, history of alcohol or drug dependence was ruled out thorough clinical interview and the German version of the Structured Clinician Interview for DSM (SCID I) [45] for DSM-IV-TR diagnostic interview.

### Structural MRI data acquisition

Patients with and without catatonia underwent native and diffusion-weighted scanning at CIMH on a 3 Tesla MAGNETOM Prisma (whiteCAT cohort) and Trio (replication cohort) MR scanner (Siemens Medical Solutions, Erlangen, Germany) using the following (different) sequences.

*whiteCAT cohort:* Native MRI:> T1-weighted 3D magnetization prepared rapid gradient echo sequence (MP-RAGE, 192 sagittal slices, image matrix = 256 * 256 mm^2^, voxel size = 1 ×1 x 1 mm3, TR = 2300 ms, TE = 3.03 ms, TI = 900 ms, flip angle = 9°). Diffusion Imaging: DTI data are acquired using an echo planar imaging (EPI) sequence with the following parameters: 32 channel multi-array head-coil, TE/TR = 86/8400 ms, 2.0 mm isotropic resolution slice thickness, field of view (FOV) = 256*256 mm^2^, 64 slices, and 81 diffusion directions at *b*-value of 1000 s/mm^2^.

*Replication cohort:* Native MRI: T1-weighted 3D-MPRAGE sequence with the following parameters: 176 sagittal slices; image matrix = 256 * 256 mm^2^, voxel size = 1 ×1 x 1 mm3; TR = 2530 ms; TE = 3.8 ms; TI = 1,100 ms; flip angle: 7°. Diffusion Imaging: DTI data were acquired using an EPI sequence with the following parameters: 32 channel multi-array head-coil, TE/TR = 86/8400 ms, 1.7 mm isotropic resolution slice thickness, field of view (FOV) = 256*256 mm^2^, 64 slices, and 60 diffusion directions at *b*-value of 1500 s/mm^2^.

### Image Processing

Both cohorts underwent identical preprocessing procedures according to our previous study [18]. T1 images underwent skull stripping (FSL bet) [46] and bias field correction using FSL [47, 48]. DWI images underwent denoising (MRtrix dwidenoise) [49], correction for Gibbs ringing artifacts (MRtrix mrdegibbs) [50], rectification for eddy currents and head motion (FSL eddy) [51] and bias field correction (MRtrix dwibiascorrect) [52]. Following this, DWI images were rigidly aligned to MNI space using FSL FLIRT, while T1 images were registered rigidly to the DWI images. After manual inspection, only images for which the preprocessing pipeline was deemed successful were retained, resulting in 101 subjects remaining in the whiteCAT cohort and 39 subjects remaining in the replication cohort, with 3 subjects from the whiteCAT cohort and 1 subject from the replication cohort being excluded. For our analysis, maps of the FA, MD and RD were generated using dtifit (FA, MD) and fslmaths (RD). We used TractSeg to generate segmentations of the 15 neurobiologically plausible tracts which represent important connections between psychomotor regions [CC, CST, T_PREM, ST_FO and ST_PREM)]. TractSeg [25, 26] is a U-Net based Convolutional Neural Network trained on data from 115 subjects of the Human Connectome Project (HCP). Its training involved the meticulous extraction of white matter (WM) tracts using a semi-automated pipeline applied to the HCP dataset. Subsequently, the model was trained to accurately predict binary masks representing 72 WM tracts based on references from the HCP. Furthermore, TractSeg has the capability to predict the start and end regions for these masks. These predictions, along with the complete binary masks, are utilized in region-of-interest (ROI)-based tracking to generate bundle-specific tractograms. TractSeg has been widely adopted in studies on mental [18, 53] and neurological [54, 55] disorders to predict binary masks for specific WM tracts. The bundle specific tractograms can then be used for further analyses. For Tractometry, various diffusion metrics (e.g. Fractional Anisotropy - FA) along the course of WM fiber tracts are extracted. This allows to quantify microstructural features of WM and potentially detect – even subtle - abnormalities. For the classification experiments, we standardized the parameter map values within the brain mask for both cohorts using z-score normalization, to eliminate biases caused by variations in the data distributions.

Finally, tractometry features were derived using TractSeg, generating bundle-specific tractograms and evaluating FA, MD, and RD along 100 points of each tract. Statistical analyses were performed pointwise along these 100 points, using an uncorrected confidence threshold of p < 0.05. Corrected p-values were aligned with the multiple comparison correction, varying for each bundle based on its correlation structure. This approach follows recommendation by Yeatman et al. [56], who argue that due to the high correlation between neighboring points on the tract profile, each point should not be treated as an independent comparison, rendering Bonferroni correction overly conservative. Instead, a permutation-based multiple comparison correction, as described by Nichols and Holmes, is applied in TractSegs tractometry pipeline, adjusting p-values appropriately considering the data’s correlation structure (with n = 5000 repetitions). Last but not least, we controlled for covariates by regressing them out before statistical analyses.

### Data analyses

#### TBSS

To detect global patterns of WM variations, TBSS was applied to each cohort separately. First, we generated the dMRI measures from the preprocessed DTI data in the subjects’ native space (using dtifit). We then executed the standard TBSS pipeline in FSL. Using TBSS, a common WM skeleton was derived from FA images by aligning and averaging the co-registered images. Subsequently, all dMRI measures were projected onto this skeleton using the standard FSL TBSS commands. The design matrix of the general linear model was structured to identify differences in voxel-wise TBSS-specific FA among catatonic and non-catatonic patients. We used the ’randomise’ command for Monte Carlo permutation tests (n = 5000 repetitions) and a confidence threshold of p < 0.05 for the corrected threshold-free cluster enhancement (TFCE) significance maps [56]. In addition to FA measures, we applied TBSS both on RD and MD measures on the skeleton along the guidelines of FSL [23].

#### Tractometry

Tractometry features were derived using TractSeg [26], generating bundle-specific tractograms and evaluating the FA, MD and RD along 100 points of each tract. Statistical analyses were performed pointwise along these 100 points. An uncorrected confidence threshold of p < 0.05 was utilized, with the corrected p-value varying for each bundle based on its correlation structure. Covariate control involved regressing them out before conducting statistical analyses.

#### Classification

In addition to the well-established analyses of TBSS and tractometry, our study further explored a ML-based approach to distinguish between catatonic and non-catatonic patients.

For predictive ML, it is essential to create feature sets that effectively represent the data distribution on which the ML algorithm bases its decision-making process. Traditional tractometry features, which enable localized tissue analysis along tracts, rely on summary statistics of the image information and therefore potentially miss valuable information due to simplifying complex image data into scalar values. In contrast, RadTract [27] combines tractometry with radiomics - a technique that extracts numerous quantitative image features (e.g. variations in pixel intensities, textures, shapes). Calculation of tractomics features using RadTract is based on pyradiomics, a widely used open-source Python package for radiomics analysis [57].

Leveraging both, the previously generated tractometry-derived features, as well as tractomics features generated via the recently developed RadTract tool, we trained individual random forest classifiers for each tract and cohort to distinguish the two classes using leave-one-out cross-validation. The experiments were conducted using the scikit-learn library [58, 59] with default parameterization.

An important aspect of this experiment was the identification of features that generalize from the whiteCAT to the replication cohort. To this end, feature selection was performed on the whiteCAT cohort and the same features were used in the classification experiments on the replication cohort.

The feature selection process consists of two steps. In a first step, the large number of tractomics features, typically more than 1000 per tract, is reduced automatically to 100 features as described in Neher et al. [27], which is equal to the number of values in the tractometry feature space. In a second step, the 10 most important features of each set (tractometry and tractomics) were selected using the random forest’s Gini importance criterion. This further reduction of the feature space is intended to prune the less important features that might lead to overfitting to the whiteCAT cohort.

The complete feature selection process was performed for each fold of the whiteCAT experiment individually, followed by a retraining of the classifier on the final 10 features. The features for the replication cohort experiment were selected by aggregating the feature importance of all folds of the whiteCAT experiment and successively selecting the ten most important features.

This method facilitated the discovery of potential biomarkers unaffected by the domain shift caused by the different imaging parameters used for the whiteCAT and the replication cohort, respectively.

### Statistical analyses

The following statistical tests were used for the TBSS and Tractometry analyses: First, in the between-group Analysis of Covariances (ANCOVA) of the whiteCAT cohort, we controlled for education, PANSS total score and SAS total score, because the two study groups differed significantly in these variables and both psychopathological symptoms and parkinsonism can modulate the severity of catatonic signs. Second, in the between-group ANCOVA of the replication cohort, we controlled for PANSS total score and SAS total score, because the two study groups differed significantly in these variables. Despite group differences in AIMS and BARS scores, we refrained from including both as covariates because catatonia signs can be distinguished from dyskinesia and akathisia by a thorough clinical examination. A modulation of the severity of catatonia signs is unlikely.

Third, employing the dimensional approach in catatonia patients (conducting a two-tailed partial correlation between tractomics FA features and NCRS and BFCRS), we controlled for age, sex, SAS, and PANSS total score, as well as MED for the whiteCAT and OLZe for the replication cohort, respectively. Further, we also followed the recommendations of the neuroimage community [60] and previous MRI studies on catatonia [18, 19, 34, 61]. Finally, p values of the identified associations in the Tractometry analyses were corrected for the number of clinical assessments in our main analysis using the Bonferroni method. For this reason, the corrected threshold was set to p = 0.01 [α = 0.05/4 tests (4 NCRS scores x 1 BFCRS total score x 1 DTI measure)].

Finally, we calculated the effect size (the Cohen’s d) and the statistical power (using G*Power) of the significant results (for results see Supplementary Material).

For completeness, a (two-sided) partial correlation was performed to determine the association between daily medication (OLZe and FLXe) and catatonia signs (motor, affective, behavioural and total NCRS scores) and the FA of CC_1, CC_3, CC_4, CC_5 and CC_6 in catatonia patients of both cohorts, controlling for age and sex.

## Results

### Clinical and demographic characteristics

Demographic and clinical characteristics of the study cohorts are shown in Tables 1 and 2. Further, in the WhiteCAT cohort, we found a significant correlation between BFCRS_total and NCRS_total scores (p = 0.023).

**Table 1:** Demographic and clinical variables of the whiteCAT cohort.

	Patients with catatonia (n = 45)	Patients without catatonia (n = 56)	t	D	Sig. (2-tailed)
Age	37.58 ± 14.54	35.62 ± 12.39	0.73	99	0.4679
Sex (m / w)	30.00/15.00	30.00/26.00		1	7.33
Education	12.98 ± 2.73	14.18 ± 2.89	−2.13	99	**0.0359**
OLZe	9.67 ± 10	8.81 ± 10.63	0.42	99	0.6774
FLXe	21.81 ± 25.68	20.93 ± 25.34	0.17	99	0.8646
PANSS_total_	70.07 ± 14.74	56.16 ± 14.82	4.70	99	**<0.001**
PANSS_positive_	14.49 ± 6.09	12.50 ± 5.04	1.80	99	0.0755
PANSS_negative_	19.36 ± 6.63	13.71 ± 5.73	4.59	99	**<.0.001**
PANSS_global_	36.22 ± 7.17	29.95 ± 7.29	4.33	99	**<.0.001**
BPRS_total_	42.82 ± 9.74	35.00 ± 9.56	4.05	99	**<.0.001**
GAF	43.00 ± 10.65	52.96 ± 14.15	−3.92	99	**<.0.001**
CGI-S	3.84 ± 1.92	2.98 ± 1.58	2.48	99	**0.015**
NCRS_motor_	2.53 ± 2.96	0.27 ± 0.65	5.57	99	**<.0.001**
NCRS_affective_	4.62 ± 2.53	1.84 ± 1.56	6.80	99	**<.0.001**
NCRS_behavioral_	3.62 ± 1.84	1.36 ± 1.20	7.46	99	**<.0.001**
NCRS_total_	10.78 ± 4.96	3.46 ± 2.42	9.70	99	**<.0.001**
BFCRS_total_	3.09 ± 2.35	0.79 ± 1.25	6.31	99	**<.0.001**
SAS	5.11 ± 3.65	1.77 ± 1.33	6.35	99	**<.0.001**
AIMS	3.84 ± 4.49	1.25 ± 1.97	3.89	99	**<.0.001**
BARS_global_	0.89 ± 1.01	0.38 ± 0.62	3.15	99	**0.002**

The t and p values (2-tailed) were obtained using an independent samples t-test between patients with and without catatonia. The p values for distribution of sex were obtained by chi-square test.

*OLZe* Olanzapine equivalents, *FLXe* Fluoxetine equivalents, *PANSS* Positive and Negative Syndrome Scale, *BPRS* Brief Psychiatric Rating Scale, *GAF* Global Assessment of Functioning, *CGI-S* Clinical Global Impression Schizophrenia, *NCRS* Northoff Catatonia Rating Scale, *BFCRS* Bush Francis Catatonia Rating Scale, *SAS* Simpson and Angus Scale, *AIMS* Abnormal involuntary movements scale, *BARS* Barnes Akathisia Rating Scale.

**Table 2:** Demographic and clinical variables of the replication cohort.

	Patients with catatonia (n = 13)	Patients without catatonia (n = 26)	t	D	Sig. (2-tailed)
Age	36.54 ± 12.07	36.85 ± 9.81	0.26	37	0.9324
Sex (m / w)	8.00/5.00	10.00/16.00		1	1.86
Education	13.46 ± 2.37	13.00 ± 3.36	0.66	37	0.6609
OLZe	14.54 ± 8.42	14.56 ± 10.52	−0.01	37	0.9956
FLXe	-	-	-	-	-
PANSS_total_	78.92 ± 21.70	55.46 ± 20.50	2.96	37	**<0.001**
PANSS_positive_	16.62 ± 9.29	12.23 ± 5.61	1.87	37	0.0739
PANSS_negative_	23.31 ± 8.13	13.81 ± 6.67	2.93	37	**0.001**
PANSS_global_	39.00 ± 12.14	29.54 ± 9.81	2.82	37	**0.013**
BPRS_total_	41.62 ± 16.39	31.58 ± 13.32	1.86	37	**0.047**
GAF	67.69 ± 14.81	74.96 ± 15.88	−1.38	37	0.1767
CGI-S	4.23 ± 0.93	3.69 ± 1.12	1.49	37	0.1445
NCRS_motor_	2.77 ± 1.64	0.31 ± 0.55	6.73	37	**<0.001**
NCRS_affective_	3.15 ± 1.77	0.62 ± 1.02	5.61	37	**<0.001**
NCRS_behavioral_	2.62 ± 1.26	0.54 ± 0.90	5.61	37	**<0.001**
NCRS_total_	8.54 ± 2.67	1.09 ± 1.62	20.23	37	**<0.001**
SAS	5.31 ± 2.75	3.00 ± 2.77	2.19	37	**0.019**
AIMS	2.85 ± 4.06	0.62 ± 2.37	1.80	37	**0.036**
BARS_global_	1.77 ± 1.79	0.69 ± 1.12	2.26	37	**0.027**

The t and p values (2-tailed) were obtained using an independent samples t-test between patients with and without catatonia. The p values for distribution of sex were obtained by chi-square test.

*OLZe* Olanzapine equivalents, *FLXe* Fluoxetine equivalents, *PANSS* Positive and Negative Syndrome Scale, *BPRS* Brief Psychiatric Rating Scale, *GAF* Global Assessment of Functioning, *CGI-S* Clinical Global Impression Schizophrenia, *NCRS* Northoff Catatonia Rating Scale, *SAS* Simpson and Angus Scale, *AIMS* Abnormal involuntary movements scale, *BARS* Barnes Akathisia Rating Scale.

### TBSS

Calculated ANCOVA’s did not identify any significant differences between both groups. However, there was a FA reduction in catatonia compared to non-catatonia patients in both cohorts (whiteCAT: p = 0.13.; replication cohort: p = 0.16). In the whiteCAT cohort, catatonia patients showed nominally lower MD values when compared to non-catatonic subjects (p = 0.11). In the replication cohort, catatonia patients showed nominally higher MD values when compared to non-catatonic subjects (p = 0.50). Finally, RD was found to be higher in catatonia patients compared to non-catatonia patients in both cohorts (whiteCAT: p = 0.09; replication cohort: p = 0.22).

### Tractometry

**Fig. 1:**
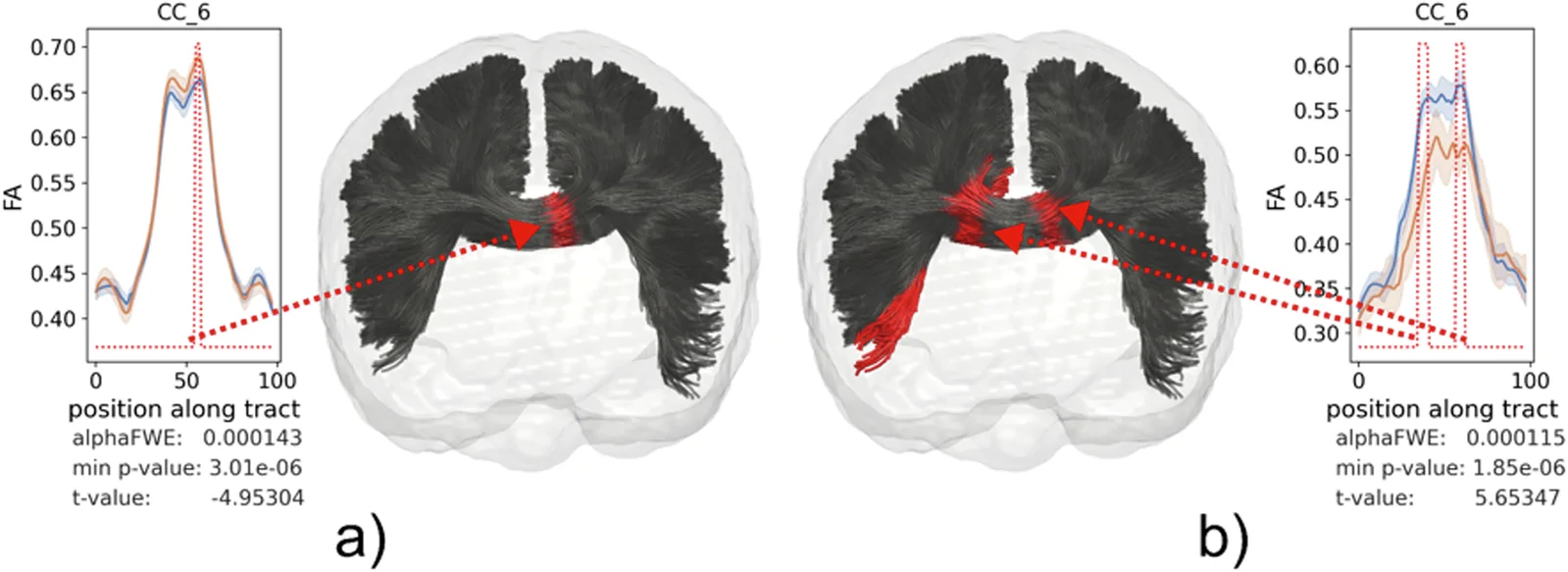
Between-group differences in white matter. Significant differences (areas highlighted in red) in FA values of CC_6 in both cohorts along the tract (**a** whiteCAT cohort; **b** replication cohort).

We found significant FA, MD and RD differences in the CC_1, CC_3, CC_4, CC_5 and CC_6 between catatonia and non-catatonia patients in both cohorts (all p-values < 0.05; s. Figure 1 and Table 3 for details).

**Table 3:** Significant differences between catatonia and non-catatonia patients found in different DTI modalities (FA, MD and RD) of the Corpus Callosum (CC) in the whiteCAT and replication cohort.

Cohort	Tract	DTI modality	Difference
**WhiteCAT cohort**	**CC_6**	**FA**	**Catatonia > non-catatonia**
WhiteCAT cohort	CC_4	FA	Catatonia > non-catatonia
WhiteCAT cohort	CC_1	MD	Catatonia < non-catatonia
WhiteCAT cohort	CC_3	MD	Catatonia < non-catatonia
**Replication cohort**	**CC_6**	**FA**	**Catatonia < non-catatonia**
Replication cohort	CC_5	FA	Catatonia < non-catatonia
Replication cohort	CC_5	RD	Catatonia > non-catatonia

*CC* Corpus Callosum, *FA* Fractional Anisotropy, *MD* Medial Diffusivity, *RD* Radial Diffusivity.

### Classification

**Fig. 2:**
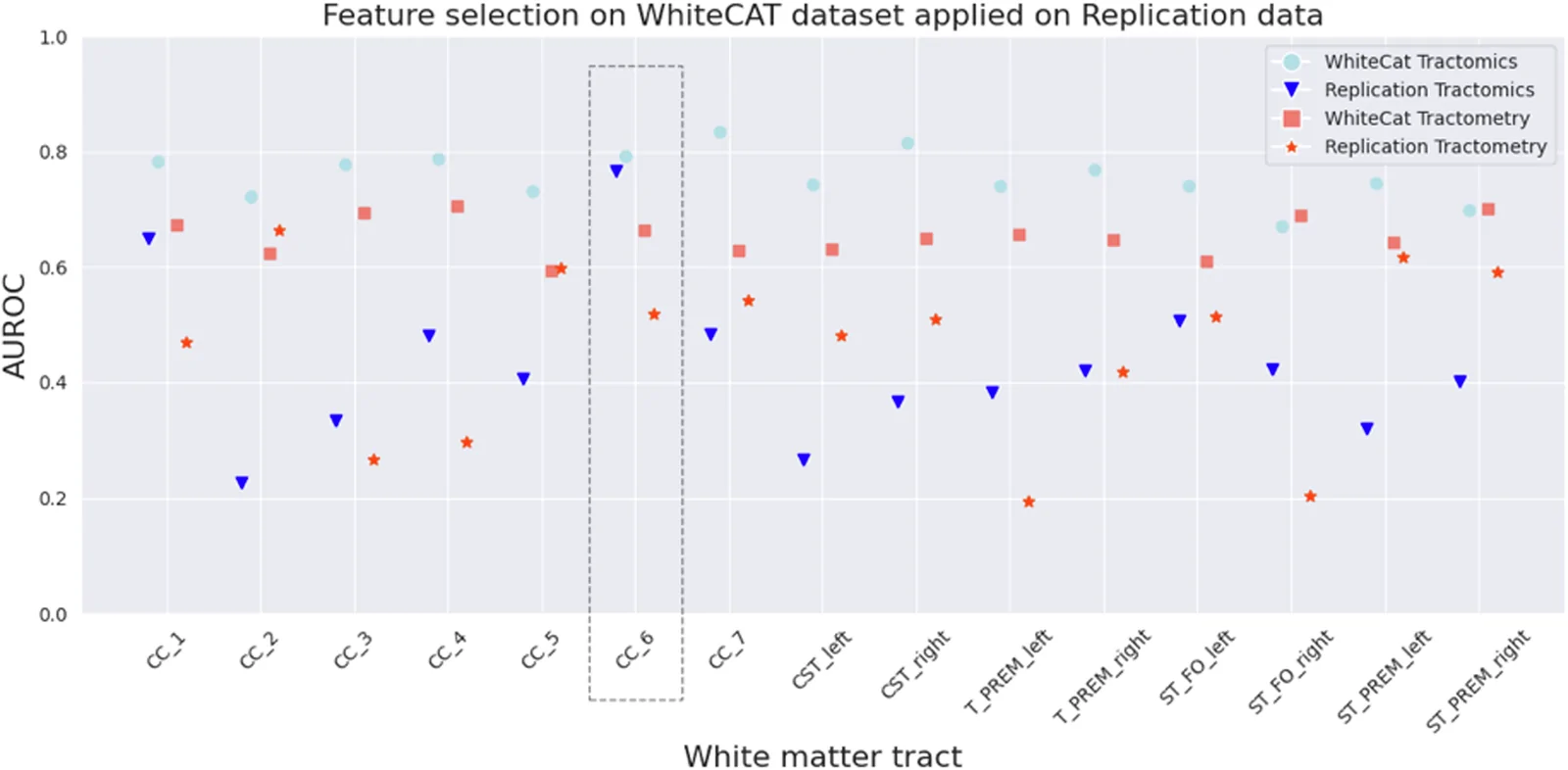
Per-tract classification results. The classifier's performance in CC_6 remained consistent across both cohorts. Additionally, in the whiteCAT cohort, tractomics demonstrated superior classification accuracy compared to tractometry features for all tracts, except for ST_PO_right.

In our classification experiments, we assessed the performance of the random forest for each tract using the area under the receiver operating characteristic curve (AUROC), as depicted in Fig. 2. Within the whiteCAT cohort, training on tractomics features exhibited superior performance compared to training on tractometry features for 13 out of the 15 tracts, while yielding comparable results for both features spaces for the remaining two tracts. Conversely, when applying the classifier with identical features to the replication cohort, the performance showed a decline across most tracts, regardless of the chosen feature space. In the replication cohort, the classifier trained on tractometry features outperformed tractomics features for seven out of the 15 tracts, whereas the reverse was observed for five tracts and equal performance was achieved on two remaining tracts.

Notably, only for the FA values of the CC_6, which had previously exhibited significant differences in the tractometry experiments for both cohorts, we were able to effectively select tractomics features from the whiteCAT cohort and apply these same features to train the classifier on the replication cohort’s, resulting in similar performances (AUROC of 0.79 for the whiteCAT data and an AUROC of 0.76 for the replication cohort). In comparison, the classifier trained on the tractometry features resulted in worse performance (AUROC of 0.66 for the whiteCAT data and 0.51 for the replication cohort). The differences in ROC curves between tractomics and tractometry features for both cohorts were assessed using the DeLong test, resulting in non-significant p-values of 0.29 for the whiteCAT cohort and 0.28 for the replication cohort.

### WM metrics vs. catatonia signs (dimensional approach)

#### TBSS

We found no significant association between NCRS scores and FA, MD or RD (voxel-wise TBSS) neither in the whiteCAT nor in the replication cohort (all p values  >  0.05). In the whiteCAT cohort, there was also no significant association between BFCRS total score and FA, MD or RD (voxel-wise TBSS).

#### Tractometry

**Fig. 3:**
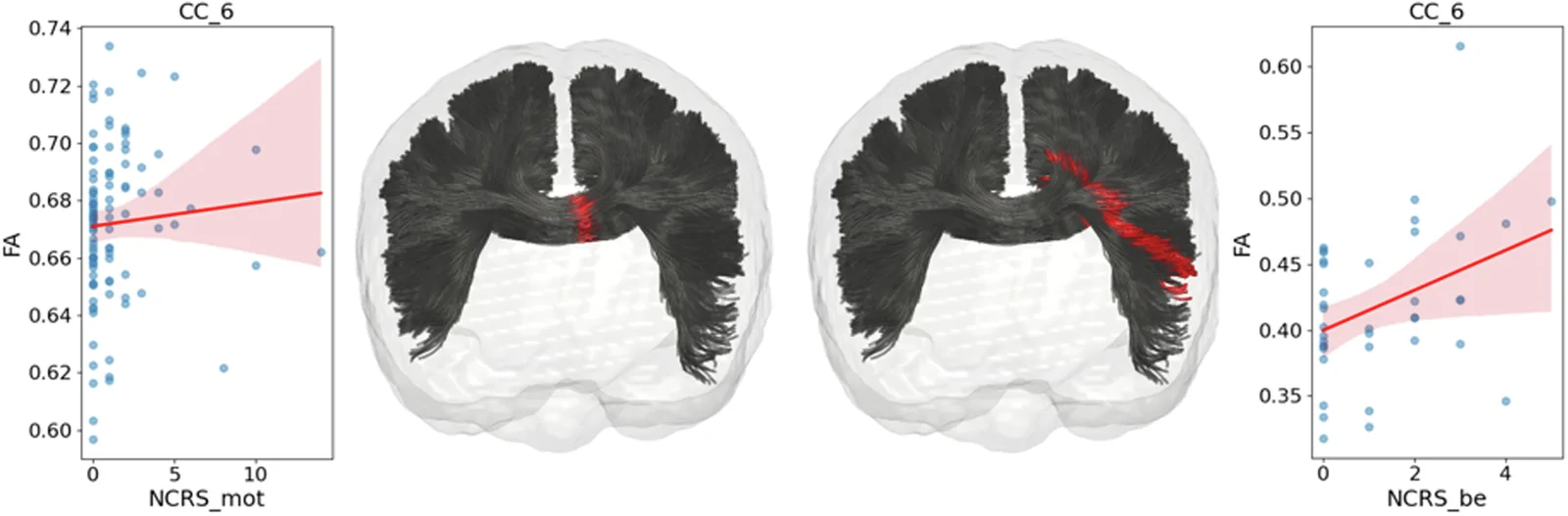
Relationship between white matter and catatonia signs. Location of significant correlation between FA values and NCRS motor (NCRS_mot) in catatonia patients of the whiteCAT cohort (left) and between FA and NCRS behavioral (NCRS_be) in catatonia patients of the replication cohort (right).

In the whiteCAT cohort, we found significant positive associations between the FA of CC_6 and NCRS motor (p = 0.006; Fig. 3) and total (p = 0.007) score. For the replication cohort we found significant positive associations between the FA of CC_6 and NCRS behavioral (p = 0.002; Fig. 3) and total (p = 0.03) score. The correlation with BFCRS total score did not reach statistical significance (min p = 0.13). All but one correlation (NCRS total score vs. FA of CC_6) did survive the Bonferroni correction for multiple testing (p < 0.01).

#### Tractomics

**Fig. 4:**
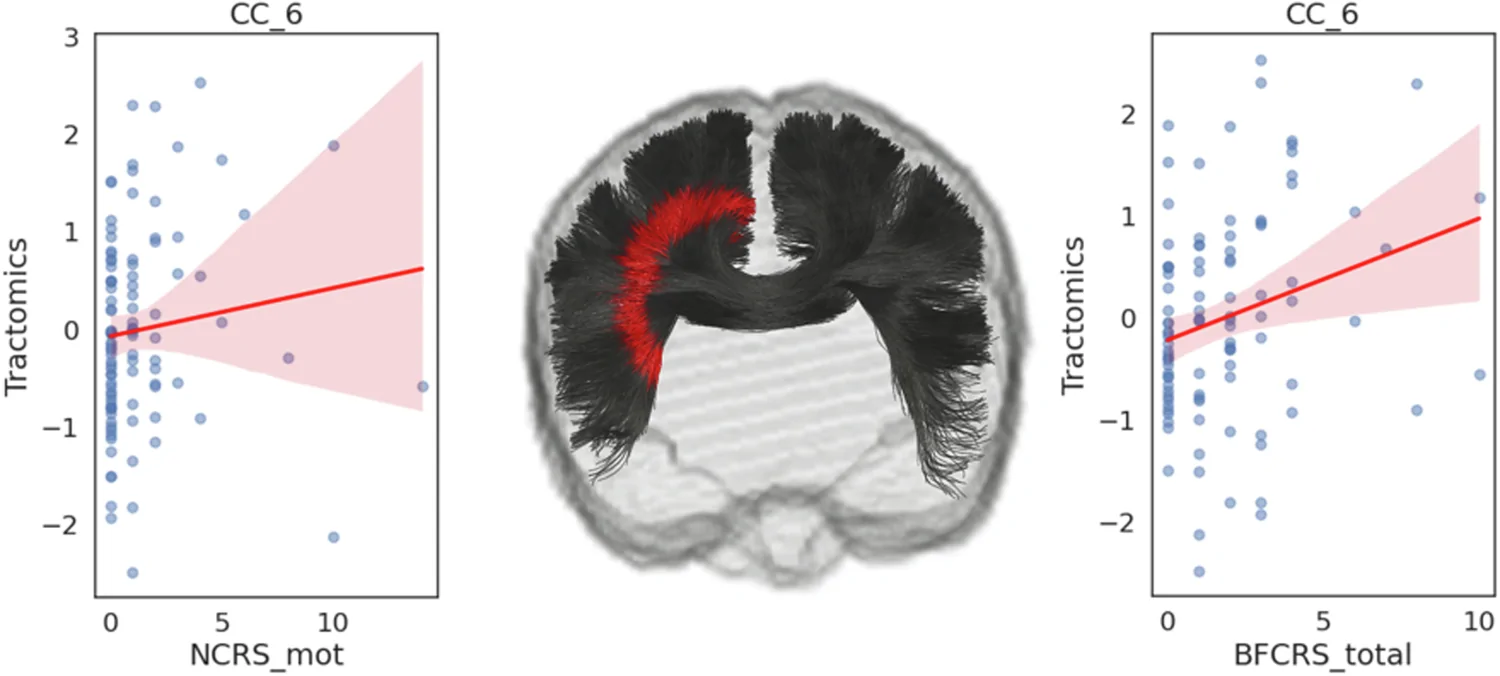
Relationship between white matter and catatonia signs. Location of significant correlation between tractomics and NCRS motor (NCRS_mot; left side) and BFCRS total score (BFCRS_total; ride side) in catatonia patients of the whiteCAT cohort.

In the whiteCAT cohort we found significant positive associations between tractomics of CC_6 and NCRS motor (p = 0.03; Fig. 4) and total (p = 0.04) score. For BFCRS total score we found a significant correlation in the CC_6 (p = 0.019; Fig. 4). In the replication cohort, no significant correlation between NCRS scores and the selected features were found. None of the correlations did survive the Bonferroni correction for multiple testing (p < 0.01).

Finally, in the whiteCAT cohort, there was a significant association between FLXe and NCRS_mot score (p = 0.03). In the replication cohort, we found no significant association between OLZe and NCRS scores. In the whiteCAT cohort, we found a correlation between FLXe and FA in CC_6 (p = 0.02). In the replication cohort, there was no significant association between OLZe and CC_6 FA values. None of the correlation did survive Bonferroni correction for multiple testing (p < 0.01).

## Discussion

This is the first and largest dMRI study that investigated microstructural WM abnormalities in two independent cohorts of catatonia patients according to ICD-11. Four main findings emerged: First, catatonia patients showed FA alterations in the CC_6 when compared to patients without catatonia across both cohorts. Second, using tractomics features generated by the newly developed tool RadTract [27], we were able to classify the patients to the correct diagnostic group based on an identical subset of CC_6 tractomics features in both cohorts with an AUROC of 0.79 whiteCAT and 0.76 for the replication cohort. Third, using RadTract, we identified potential catatonia-specific imaging biomarkers in WM tracts connecting psychomotor regions which outperformed traditional tractometry biomarkers on the whiteCAT cohort and this result is therefore in line with the original RadTact publication [27]. Fourth, we found a significant correlation between FA of CC_6 (TractSeg and RadTract) and catatonia signs assessed with NCRS and BFCRS in both cohorts.

The first finding of this study is interesting for a number of reasons: Firstly, standard voxel-based TBSS was unable to detect significant variations in FA between catatonic and non-catatonic individuals in both cohorts. This finding corroborates the study by Wasserthal et al. [18] and we might speculate that the WM abnormalities associated with catatonia were not sufficiently severe to be identified by using voxel-based TBSS. Instead, precise dMRI methods, including individual WM tract segmentation (TractSeg) and rich, informative data representations of these tracts (RadTract), may be essential to identify WM abnormalities associated with catatonia. Secondly, CC serves as one of the most prominent tracts in the human brain that is responsible for the mediation of interhemispheric transfer in terms of increased inhibition or reduced facilitation [62]. That said, CC facilitates interhemispheric communication, allowing for the integration of cognitive, emotional, and sensory information between brain regions [22]. Furthermore, intact transcallosal functioning is essential for sustained attention, motor control, and synchronization of bilateral movements [63]. Third, structural and functional alterations of the CC can lead to deficits in information sharing between brain hemispheres, potentially contributing to cognitive impairments, emotional dysregulation, and social difficulties in patients with psychiatric disorders [64]. For instance, a recent study by Piras et al. [65] found that CC undergoes significant alterations in individuals with obsessive-compulsive disorder, exhibits specific changes in those with SSD, and remains unaffected in individuals with bipolar disorder (BD) and major depressive disorder. An earlier mega-analysis by Koshiyama et al. [66] found that SSD, BD, and autism spectrum disorder patients exhibit comparable variations in the microstructure of WM in the CC. Overall, these findings illuminate the similarities and differences in the CC among various mental disorders, offering valuable insights into the role of this major brain fiber tract in the pathophysiology of each specific mental illness. Fourth, animal studies (for review see Mallien et al. [67]), case reports and dMRI studies have found a relation between catatonia and structural changes in the CC. For instance, Poggi et al. [68] found a very subtle hypomyelination in the prefrontal CC of *Mbp*^*+/‐*^ (heterozygous) mice (MBP=myelin basic protein) to be associated with age‐related catatonia-like behavior. Further, Arora and Praharaj [69] described the occurrence of catatonia in a patient with a butterfly glioma that originated from the CC and had bilateral medial frontal extensions. Palm et al. [70, 71] presented a 35-year-old catatonia patient with isolated CC aplasia who did not respond to initial therapy with lorazepam, but improved significantly after a sequence of unilateral electroconvulsive therapy (ECT). Due to the severity and relapses of catatonic episodes, this patient received weekly ECT sessions for three years [72]. A more recent case report by Garg et al. [73] described a 28-year male patient with Shapiro syndrome (a triad of CC agenesis, episodes of hyperhidrosis, and hypothermia) presenting with catatonia. Other case reports have reported catatonic signs (e.g. poverty of speech, immobility, rigidity, psychomotor retardation, blank expressions, withdrawn) in Shapiro syndrome [74, 75], but have not assigned a diagnosis of catatonia to these patients. Furthermore, three out of four previous dMRI studies showed CC alterations in catatonic patients [18, 19, 21]. Viher et al. [19] detected a group effect of WM within the CC. In the study by Wasserthal et al. [18], we used tractometry and found reduced FA in CC in patients with catatonia compared to patients without catatonia according to NCRS criteria. Similarly, the study by Krivoy et al. [21] found that SSD patients with catatonia exhibited notably higher FA in the splenium of the CC compared to those without catatonia. Our results also reflect the inconsistencies in the current evidence since in the whiteCAT group catatonia patients showed reduced FA compared to the control group, it was exactly the opposite in the replication cohort. Variations in FA are not uncommon and may be due to state-dependent psychopathology [76]. That said, the whiteCAT cohort showed higher NCRS total scores and different symptom patterns (higher NCRS affective scores) than the replication cohort. Therefore, we speculate that different symptom patterns across the three catatonia domains (within-group variability) might lead to different changes in the WM microstructure (e.g. reduced myelination, axonal density or altered fibre orientation) and hence, manifest as FA fluctuations. This is also in line with previous studies on SSD and mood disorders patients that showed inconsistent results, with some studies reporting decreases in FA, while others report increases or no significant differences [77]. Taken together, based on scientific evidence [78] and this study, WM microstructural alterations in the CC (both decreased and increased and alone or in combination with other alterations) can lead to sudden affective, motor and behavioral signs and the manifestation of catatonia in specific individuals.

The second main finding is of importance in the field of neuroimaging research, because by leveraging tractomics in the CC, clinicians and researchers could potentially identify distinct neurobiological signatures associated with catatonia. This is even more remarkable considering that we successfully distinguished between two distinct groups of patients with mental disorders, not just catatonia vs. healthy subjects. The fact that we achieved high AUROC in distinguishing groups of catatonia vs. non-catatonia patients thus is a major achievement which, as far as we know, has not been achieved in current neuroimaging. This speaks for the importance and validity of our WM markers, notably CC_6.

However, there have been, only very few studies to date that have attempted to use ML approaches to classify psychiatric patients into the correct diagnostic group. The study of Deng et al. [79] used a random forest algorithm on tractography-based diffusion characteristics to distinguish patients with first-episode schizophrenia from healthy persons. The classification process involved the utilization of recursive feature elimination to identify the final WM characteristics. Using the WM characteristics of inter-hemispheric fibres, the cerebello-thalamo-cortical circuits, and the long association fibres, patients were differentiated from healthy individuals with an accuracy of 71.0%. The sensitivity was 67.3%, the specificity was 75.0%, and the AUROC was 79.3% (χ2 p < 0.001). During the validation process, when 20% of the sample was withheld, patients were differentiated from healthy persons with an overall accuracy of 76.0%. The sensitivity was 76.9%, the specificity was 75.0%, and the AUROC was 73.1% (χ2 p = 0.012). Another study by Kenney et al. [80] investigated neuroanatomical markers of psychotic experience (PE) in early and later phases of adolescence using ML. Logistic regression with Elastic Net regularization was utilized to apply machine learning to T1-weighted and dMRI data. The objective was to categorize teenagers with subclinical psychotic episodes compared to controls at 3 different timepoints. This study found that individuals who had PE in the future between the ages of 18 and 20 were most effectively differentiated from the control group by analyzing certain brain areas, including the CC. Furthermore, the authors concluded that young individuals with PE exhibit accelerated advancement of WM microstructural alterations in the right CC. A recent study by Saglam et al. [81] found that ML models, using FA values of CC reconstructed through global probabilistic tractography, can differentiate between early-onset schizophrenia and early BP. WM analysis techniques together with ML approaches can not only enhance diagnostic accuracy but also have far-reaching implications for reducing misdiagnosis, improving patient outcomes, and advancing our understanding of the neurobiology underpinning psychiatric disorders, ultimately paving the way for more targeted and effective treatments in the realm of mental health. These studies, including our work, highlight the potential of employing ML and feature selection techniques in exploring potential biomarkers. Notably, our study distinguishes itself by utilizing an independent replication cohort to assess the stability of selected features and mitigate potential overfitting associated with the extensive numbers of features.

The third finding underscores the potential of introducing more extensive sets of biomarkers to enhance the classification of patients with psychiatric disorders, leveraging disease-specific WM alterations. By extracting a wider spectrum of quantitative features from WM tracts, tractomics enables a more objective and data-driven assessment of WM microstructural changes within catatonia-specific tracts, in contrast to tractometry. This capability can enhance diagnostic accuracy by detecting subtle WM alterations that traditional qualitative DTI methods may not discern. However, further advancements beyond tractometry or tractomics are necessary to adequately distinguish mental disorders such as catatonia.

The fourth finding is consistent with the first and once again suggests that disruptions in interhemispheric connectivity and information exchange within the CC may contribute to the complexity and intensity of catatonia signs [12, 18]. Since the CC facilitates communication between the left and right hemispheres of the brain, changes in FA of CC_6 may affect the connectivity of psychomotor-related brain regions, contributing to the severity and complexity of psychomotor signs in catatonia. However, our finding is unique, as previous dMRI studies on catatonia found neither significant association between FA of the CC and severity of catatonia signs nor examined this relationship at all [12]. For instance, using TBSS, Viher et al. [19] found no association between FA of CC and BFCRS. Further, although the study by Wasserthal et al. [18] used three different methods, no association was found between WM in CC and NCRS scores. Finally, Krivoy et al. [21] did not analyze the relationship between the severity of catatonia and the FA values at all, because the patients were in remission during MRI and the BFCRS scores were 0 in both groups.

Furthermore, catatonia patients have markedly different GAF scores [82, 83] possibly due to different impact of catatonia signs on their daily activities and overall well-being [84, 85]. Bräunig et al. [82] found that catatonia patients with mania experienced a higher frequency of mixed episodes, more severe manic symptoms, increased levels of general psychopathology, a greater likelihood of having additional medical conditions, longer hospital stays, and lower GAF scores compared to those without catatonia signs. In a more recent study by Nadeselingam et al. [83], catatonia patients had lower scores on the GAF and SOFAS measures in comparison to those without catatonia. This is also supported by findings of Kline et al. [86] who reported lower GAF scores in catatonia and suggested that catatonia could be associated with higher symptom burden as well as increased negative affect compared to patients without catatonia. However, global functioning maintained in catatonia patients in the replication cohort and hence, this might reflect better preservation or resilience of WM integrity. This finding suggests possible protective effects against more severe WM abnormalities among those with lower GAF scores leading to different directions of WM alterations between the two cohorts (Table 3).

Interestingly, our TBSS analysis did not reveal any statistically significant differences in FA—an indicator of both myelination and axonal integrity [87–90]—between individuals with and without catatonia. Similarly, no significant correlations were found between changes in FA and catatonia signs. These findings align with the study by Wasserthal et al. [18], which also reported a lack of significant FA differences (based on TBSS) in SSD patients with and without catatonia when compared to healthy controls. This recurring outcome across different studies highlights the inherent challenges of detecting WM alterations in mental disorders, where differences can be subtle and complex. TBSS remains a widely respected method for analyzing WM integrity and is often regarded as a gold standard in the field [24, 91]. However, its sensitivity may be limited when studying complex mental disorders, where between-group differences can be harder to detect compared to studies involving clear-cut neuropsychiatric conditions and healthy controls. But, the absence of significant findings in our study and others [92–94] should not be seen as a limitation of TBSS itself, but rather as a reflection of the inherent difficulties in detecting subtle variations in WM among individuals with phenomenologically similar and complex mental disorders. While some concerns have been raised regarding the anatomical specificity of TBSS, particularly in areas where multiple fiber tracts converge (e.g., the superior projections of the corpus callosum and the corona radiate), TBSS also faces criticism for its potential lack of tract-specificity (see Bach et al. [95] for details). This is primarily due to the skeletonization process, which relies solely on FA values and discards crucial orientation information from the diffusion data, potentially causing neighboring tracts with similar FA values to collapse onto each other. Nevertheless, TBSS remains a robust and well-validated approach for group-level comparisons [95]. To explore alternative approaches, we used TractSeg [25, 26], a novel method based on convolutional neural networks that segments tracts using the fiber orientation distribution function (fODF) peaks without relying on tractography or parcellation. TractSeg offers certain advantages, such as avoiding some of the anatomical specificity issues associated with TBSS. However, it is important to acknowledge that TractSeg is not immune to limitations, including the potential for false positives. False positive results in TractSeg may arise due to noisy or low-quality DTI data affecting fiber orientation estimation, challenges in distinguishing complex WM anatomy, variability in data acquisition across different datasets, and the limitations of neural network predictions which can overfit to certain features. These factors can lead to over-segmentation or incorrect identification of fiber tracts, particularly in regions where fiber pathways converge or are difficult to resolve. Moreover, tractometry, the process of quantifying properties along tracts, also comes with its sensitivity to the accuracy of tract segmentation and can be affected by variability in tract profiles across individuals. Therefore, while TractSeg provides an alternative, tract-specific perspective on WM analysis, we emphasize that both TBSS and TractSeg have their own strengths and limitations, and more research is needed to fully understand their comparative performance in patients with mental disorders.

The question now arises as to how we can integrate the above results into the previous catatonia models? Since its initial clinical description in 1874, catatonia has been regarded from two distinct clinical and neurobiological perspectives: (1) as an exclusively motor disorder (Kraepelin and Bleuler’s legacies) and (2) as a psychomotor/affective disorder (Kahlbaum’s legacy) (for details see [96]). In the last two decades, neuroimaging research into the pathophysiological mechanisms of catatonia further validated this historical distinction [18, 19]. MRI studies employing motor/behavioral rating scales/criteria such as BFCRS (motor approach) have postulated that the neuronal and biochemical underpinnings of catatonia are alterations in dopamine-mediated cortical and subcortical motor regions [12]. On the contrary, MRI studies employing the NCRS (psychomotor approach) identified aberrant networks in the higher-order frontoparietal region that lacked adequate modulation through the transmission of glutamate and gamma-aminobutyric acid (GABA) [12]. It is crucial to note that the overwhelming majority of case-control investigations pertaining to the pathogenesis of catatonia have been carried out on individuals who suffered from catatonia associated with another mental disorder [97]. From an anatomical point of view, the prefrontal, premotor, and supplementary motor cortical regions are responsible for the compartments of the anterior third of the CC, which include the rostrum, genu, and rostral body [98]. Fibers originating in the motor cortex are believed to cross the CC via the anterior midbody, whereas somaesthetic and posterior parietal fiber bundles cross through the posterior midbody [98]. The posterior third of the CC, which includes the isthmus and splenium, is allocated to the temporal, parietal, and occipital cortical areas [98]. In the present study, we found FA alterations in anterior, midbody and posterior parts of CC. This said, CC is responsible for interhemispheric communication and FA changes in all segments can disrupt inter-hemispheric transfer between both motor- and psychomotor-associated regions. These changes might lead to very heterogeneous catatonia signs and hence, the present findings fit very well with the previous results of both motor and psychomotor approaches.

### Strengths, limitations and methodological considerations

The study sample size, two well-matched patients cohorts, use of ICD-11 catatonia criteria and the comprehensive set of 3 sophisticated analysis methods (incl. ML) are strengths of our study. However, some methodological aspects limit the generalizability of our results: First, a potential limitation of this study could be the absence of healthy controls; however, since the objective was to identify catatonia-related WM microstructural alterations, psychiatric patients without catatonia serve as a valid control group. In line with this, another possible limitation is that this study examined solely patients with catatonia associated with another mental disorder as per the ICD-11 criteria (6A40) and did not include other ICD-11 catatonia categories: Catatonia induced by substances or medications (6A41), Secondary catatonia syndrome (6E69), and Catatonia, unspecified (6A4Z). Consequently, our findings might not reflect the full spectrum of catatonia pathophysiology, potentially limiting the generalizability of the results to all catatonia variants. Second, although we controlled for medication effects, antipsychotic and antidepressant agents should be regarded as a possible confounding variable. Additionally, it is important to note that the complete record of antipsychotic or antidepressant medication usage in the current patient sample was not feasible to obtain. The examination of long-term medication effects on catatonia and WM is particularly reliant on the cumulative dosage of antipsychotic and antidepressant medication. Hence, the present daily dose could not accurately depict the long-term cumulative impact of antipsychotics or antidepressants on catatonia-related networks. Third, it is not possible to make any assertions on the fluctuating nature of catatonia. The inquiry regarding the stability of signs vs their dynamics can be effectively addressed by longitudinal monitoring, particularly utilizing electronic instruments. Fourth, our DTI sequences did not encompass the brainstem and cerebellum, since we prioritized obtaining better resolution images of cortical and midline regions. Consequently, we are unable to assess the impact of WM microstructural changes in the brainstem and cerebellar tracts on catatonia. Fifth, despite representing the largest cohort in catatonia research, it remains relatively small for conducting ML experiments aimed at training models that are generalizable. This is particularly evident in the size of the replication cohort, which might be related to the non-significant results of the Delong test. The decline in the performance of the classifier across most WM tracts in the replication cohort may be due to changes in the distribution of the datasets. Recent efforts have focused on harmonizing datasets with varying dMRI acquisition protocols [99]. Better harmonized datasets could benefit the classifier, potentially yielding more generalizable features. This could hypothetically enhance performance on the replication dataset post-feature selection on a specific. Sixth, the two different cohorts were not fully independent, because they were both recruited at the same site (CIMH). Still, we consider our two cohorts to be suitable for testing our hypotheses and validating our results, because of the following reasons: (i) Both cohorts were examined during different periods of time, the whiteCAT cohort between 04/2022 and 002/2024 and the replication cohort between 02/2017 and 10/2018. (ii) The two cohorts were examined for catatonia signs by completely different psychiatrists, and the replication cohort was not originally recruited according to the ICD-11 criteria. Patients in the replication cohort were only SSD-patients, and were primarily recruited for the purpose of investigating neurobiological underpinnings of catatonia according to NCRS. (iii) The two cohorts were examined with different MRI scanners and DWI parameters, the whiteCAT cohort with 3 T Siemens PRISMA scanner and the replication cohort with 3 T Siemens Trio scanner. (iv) Large consortia such as Human Connectome Project or UK Biobank nowadays may allow for pooling over much larger numbers of patients. However, to the best of our knowledge, none of the extant neuroimaging consortia do explicitly assess catatonia signs according to ICD-11, thus limiting detailed investigation of neuronal correlates of catatonia in larger samples. Furthermore, using different MRI scanners (TRIO vs. PRISMA), DWI parameters, patient groups (transdiagnostic vs. SSD only sample), and examiners exhibiting different clinical experience across two cohorts presents advantages and challenges for replicating and validating findings in neuroimaging studies on catatonia. The diversity of equipment and settings in the AT and replication cohort enhanced the generalizability of our results and hence, we were able to show that our findings are robust across various scanners and populations. However, this variability can also introduce inconsistencies in image quality and measurement precision, particularly impacting the reliability of FA results (e.g. different direction between cohorts). Differences in patient demographics, clinical phenotypes, and examiner techniques can further complicate comparisons and introduce biases. Still, we believe our results are robust and reliable due to the rigorous methodologies employed and the consistent patterns observed across both cohorts. Nevertheless, further studies are essential to replicate our findings, which will help validate the generalizability and enhance the understanding of the observed effects in diverse catatonia groups and using various dMRI technologies.

Seventh, there is a highly significant difference in PANSS total and PANSS negative scores between the catatonia patients and controls. Psychopathological symptoms can significantly influence, and at times even mask, the psychomotor syndromes such as catatonia, particularly in patients experiencing anxiety, mania, impulsivity, anhedonia, or psychomotor slowing. Due to the intrinsic presence of these signs in mental disorders, it is challenging to completely disentangle their effects from the signs of catatonia. This overlap complicates the assessment and interpretation of catatonic features. Currently, it remains uncertain if an alternative method exists that could effectively isolate the impact of these psychopathological symptoms, as all patients with mental disorders will invariably exhibit such clinical features to some degree. This limitation underscores the complexity of diagnosing and studying catatonia across different patients’ populations.

## Conclusion

This study employed three distinct analytic methods to comprehensively assess and characterize WM microstructural alterations underlying catatonia. Through the utilization of TBSS, TractSeg, and Radtract, we were able to provide a multifaceted view of WM microstructure, revealing valuable insights into its complexity and interplay with severity of catatonia signs. These findings also underscore the potential of employing a ML approach to WM alterations in catatonia in order to elucidate the underlying pathophysiology and provide evidence for potential biomarkers.

## Supplementary information

Supplementary material
