# Supplementary material for: Deciphering white matter microstructural alterations in catatonia according to ICD-11: replication and machine learning analysis
Source: Mol Psychiatry. 2024 Dec 2;30(5):2095–107. doi: 10.1038/s41380-024-02821-0 (PMC12014485; doi:10.1038/s41380-024-02821-0)
Supplement: Supplementary file 1 — Supplementary material [file 41380_2024_2821_MOESM1_ESM.docx]

**Supplementary material**

1. **Patients’ medication**

At the time of inclusion, most patients were receiving treatment with antipsychotics and/or antidepressants according to their psychiatrists’ choice, for further information please see the table provided. Within the whiteCAT cohort, 0 catatonia patients (cat) and 3 non catatonia (non-cat) patients were not taking any medication. 86 patients (38 cat and 48 non-cat) received one antipsychotic and 45 patients (32 cat and 13 non-cat) received two antipsychotics. Of note, the number of cat patients treated with first-generation antipsychotics was very low (cat n=6, non-cat n=2).  Patients within the whiteCAT cohort were treated with amisulpride (cat n=3; non cat n=2), olanzapine (cat n=8; non cat n=2), clozapine (cat n=10, non-cat n=4), quetiapine (cat n=5, non-cat n=12), aripiprazole (cat n=6, non-cat n=8), cariprazine (cat n=4, non-cat n=2), risperidone (cat n=4, non-cat n=1), paliperidone palmitate (cat n=0, non-cat n=2). Within the whiteCAT cohort, 35 patients (15 cat and 20 non cat) received one antidepressant and 10 patients (4 cat and 6 non cat) received two antidepressants. Patients within the whiteCAT cohort were treated with sertraline (cat n=4, non-cat n=10), venlafaxine (cat n=9, non-cat n=4), citalopram (cat n=4, non-cat n=1), fluoxetine (cat n=1, non-cat n=0), bupropione (cat n=1, non-cat n=6), trazodone (cat n=1, non-cat n=3) and mirtazapine (cat n=2, non-cat n=2). 11 patients received phase prophylactics (cat n=5, non-cat n=6), one patient of the non-cat group received two phase prophylactic drugs. Within the replication cohort, 1 cat patient and 1 non-cat patient were not taking any medication. 25 patients (9 cat and 16 non-cat) received one antipsychotic and 10 patients (3 cat and 7 non-cat) received two antipsychotics. Patients were treated with amisulpride (cat n=2; non cat n=6), olanzapine (cat n=5; non cat n=5), clozapine (cat n=4, non-cat n=2), quetiapine (cat n=1, non-cat n=5), aripiprazole (cat n=2, non-cat n=6), cariprazine, risperidone (cat n=0, non-cat n=2), paliperidone palmitate (cat n=0, non-cat n=2). Within the replication cohort, one patient was treated with venlafaxine, three patients within the non-cat group were treated with phase prophylactics. One patient in each group was treated with first-generation antipsychotics.

|  | ***whiteCAT cohort*** | | ***replication cohort*** | |
| --- | --- | --- | --- | --- |
| ***Antipsychotics Second Generation*** | ***N (cat)*** | ***N (non cat)*** | ***N (cat)*** | ***N (non cat)*** |
| *Amisulpride* | *3* | *2* | *2* | *6* |
| *Olanzapine* | *8* | *9* | *5* | *5* |
| *Clozapine* | *10* | *4* | *4* | *2* |
| *Quetiapine* | *5* | *12* | *1* | *5* |
| *Aripiprazole* | *6* | *8* | *2* | *6* |
| *Cariprazine* | *4* | *2* | *0* | *0* |
| *Risperidone* | *4* | *1* | *0* | *2* |
| *Paliperidone* | *0* | *2* | *0* | *2* |
| *Ziprasidone* | *1* | *0* | *0* | *0* |
| *No medication* | *0* | *3* | *1* | *1* |
| ***Antipsychotics First Generation*** | ***N (cat)*** | ***N (non cat)*** | ***N (cat)*** | ***N (non cat)*** |
| *Haloperidole* | *2* | *0* | *0* | *1* |
| *Fluanxole* | *0* | *2* | *1* | *0* |
| *Chlorprothixen* | *1* | *0* | *0* | *0* |
| *Levomepromazine* | *1* | *0* | *0* | *0* |
| *Dipiperone* | *2* | *0* | *0* | *0* |
| ***Antidepressants*** | ***N (cat)*** | ***N (non cat)*** | ***N (cat)*** | ***N (non cat)*** |
| *Sertraline* | *4* | *10* | *0* | *0* |
| *Venlafaxine* | *9* | *4* | *0* | *1* |
| *Citalopram* | *4* | *1* | *0* | *0* |
| *Fluoxetine* | *1* | *0* | *0* | *0* |
| *Bupropion* | *1* | *6* | *0* | *0* |
| *Trazodone* | *1* | *3* | *0* | *0* |
| *Mirtazapine* | *2* | *2* | *0* | *0* |
| ***Phase prophylactics*** | ***N (cat)*** | ***N (non cat)*** | ***N (cat)*** | ***N (non cat)*** |
| *Lithium* | *3* | *2* | *0* | *1* |
| *Valproate* | *2* | *3* | *0* | *1* |
| *Lamotrigine* | *0* | *2* | *0* | *1* |

**Supplementary table 1.** Overview of antipsychotics and antidepressants taken by the patients in both cohorts.

1. **TBSS**

*2.1. Additional controlling for BARS_global*

Additional controlling for BARS_global in ANCOVA did not yield to statistical significance in the whiteCAT or replication data in FA (p=0.23; p=0,19), MD (p=0.22; p=0.39) and RD (p=0,19; p=0,19).

1. **Tractometry**

*3.1. Additional controlling for BARS_global*

Controlling for BARS_global in Tractometry did not yield additional differences in the white matter tracts for FA, MD and RD.

*3.2*. Power Analyses

We calculated the effect size (the Cohen's d) and the statistical power (using G*Power) of the significant results. First, the significant differences of the FA in the CC_6 showed Cohen's d values of -0.42 in the whiteCAT and 1.5 in the replication cohort, which represent moderate and large effect sizes. We found a statistical power of 0.3 and 0.95 (1-ß error probability).

Second, the significant correlations between FA of the CC_6 and NCRS motor and NRCRS_total score in the whiteCAT cohort showed Cohen’s d values of 0.57 and 0.56, which represent medium effect sizes. We found a statistical power of 0.77 and 0.98 (1-ß error probability) representing medium to large effect sizes, respectively. For the replication cohort the significant correlation between FA of the CC_6 and NCRS_behavirol score we found Cohens d values of 1.16 and statistical power of 0.99, representing a large effect size. The significant correlations of Tractomics features and NCRS_mot as well as BFCRS showed Cohens d of 0.44 and 0.49 representing medium effect sizes and showed statistical power of 0.88 as well as 0.93.
